# The Role of Social Support and Acculturation Factors on Postpartum Mental Health Among Latinas in the MADRES Pregnancy Cohort

**DOI:** 10.1007/s10903-023-01542-w

**Published:** 2023-10-28

**Authors:** Karina Corona, Tingyu Yang, Genevieve Dunton, Claudia Toledo-Corral, Brendan Grubbs, Sandrah P. Eckel, Jill Johnston, Thomas Chavez, Deborah Lerner, Nathana Lurvey, Laila Al-Marayati, Rima Habre, Shohreh F. Farzan, Carrie V. Breton, Theresa M. Bastain

**Affiliations:** 1 Department of Population and Public Health Sciences, Keck School of Medicine, University of Southern California, Los Angeles, CA USA; 2 Department of Psychology, University of Southern California, Los Angeles, CA USA; 3 Department of Health Sciences, California State University, Northridge, Northridge, CA USA; 4 Department of Obstetrics and Gynecology, Keck School of Medicine, Los Angeles, CA USA; 5 Eisner Health, Los Angeles, CA USA

**Keywords:** Social support, Acculturation factors, Perceived stress, Postpartum distress, Depression

## Abstract

We examined the associations between social support and postpartum mental health in 137 U.S. and foreign-born Latinas in the MADRES pregnancy cohort. We also examined whether language, years in the U.S., and country of birth moderates these relationships. Participants were administered PROMIS support measures 1 month postpartum; the Perceived Stress and Postpartum Distress Measure 3, 6, and 12 months postpartum; and the CESD scale 12 months postpartum. Perceived stress was lower at 6 months postpartum for women reporting higher emotional (*p* = 0.01), informational (*p* = 0.03), and instrumental support (*p* < 0.001); and lower at 12 months postpartum for women reporting higher emotional support (*p* = 0.01). Distress at 6 months was lower in women reporting higher emotional support (*p* = 0.03). Interactions suggest that associations were stronger for mothers that speak Spanish, spent fewer years in the U.S., and were born in Central America.

**Supplementary Information:**

The online version contains supplementary material available at 10.1007/s10903-023-01542-w.

## Introduction

Postpartum mental health among U.S.-born and foreign-born Latinas is a neglected area of study in health disparities research. While as many as one-fifth of new mothers may experience postpartum depression (PPD), defined as an episode of major or minor depressive disorder in the postpartum period, during the first three months after giving birth [1], the risk for PPD is elevated among Latinas. Approximately 43% of Latina mothers report postpartum depressive symptoms in the immediate postpartum period [2]. Adverse mental health outcomes such as postpartum depression and anxiety are associated with serious health outcomes (e.g., lower quality of life) for both mothers and their offspring (i.e., disrupted sleep patterns) [1, 3]. Evidence indicates that postpartum psychological distress (e.g., stress, anxiety, and depression) is similarly linked with infants’ poor cognitive and emotional maladjustment [4].

Several risk factors have been associated with postpartum depression including symptoms of prenatal depression, low social support, low socioeconomic status, and previous history of depression and anxiety [5]. Specifically, social support is one external factor that helps people cope with stressful life events [6]. Emotional support refers to the perception that one is cared for and valued including the provision of empathy and trust. Informational support is the perception of helpful information or advice from others during a time of stress. Instrumental support consists of tangible aid and services that are available when needed [7]. Leahy-Warren et al. [8] found that emotional, informational, and instrumental social support were inversely related to postpartum depression among first time postpartum mothers. Higher levels of general social support have been shown to buffer against postpartum depression [9].

In Latinas’ postpartum maternal health functioning, social support may be beneficial since it aligns well with Latino social norms for seeking help [10]. Greater social support has been previously associated with lower perceived stress in pregnant and postpartum Latina women [11]. Consistent with previous studies, among an ethnically diverse sample of mothers, higher postpartum social support (e.g., total, emotional, tangible, affectionate, positive social interaction, and paternal support) had a protective association with PPD [12]. Among women who received greater social support, higher levels of family stressors were associated with lower postpartum depression in a sample of Mexican American women [13]. This study was cross-sectional at 6 weeks postpartum and could not determine the directionality of social support and PPD. Further, while the study examined Black and Latina women, the study did not consider other contextual factors such as acculturation that may influence the perception of social support. Fewer studies have examined the effect of multidimensional aspects of social support (e.g., companionship, advice, and emotional support) among U.S. and foreign-born U.S., born women’s maternal mental health.

Despite extensive research focusing on PPD prevalence and risk factors in the general population, there is a lack of research examining specific cultural factors that may moderate the relationship between social support and postpartum mental health among low-income, minority Latina women [14]. An example of culturally relevant psychosocial factors that influence the Latina postpartum experience is mothers’ level of acculturation [14]. Latina immigrant women may be particularly vulnerable to serious postpartum mental health outcomes because of stigma, guilt, and healthcare access related to maternal and provider recognition of depression [15]. Foreign-born Latinas may face unique stressors related to their immigrant experience that exacerbate PPD, including documentation status, language, country of birth, and/or time spent in the U.S. [14]. There is scant research examining PPD risk and/or attenuating factors among Latinas born in and outside the U.S. including whether there are differences by country of origin. Rather, most biopsychosocial models of health tend to treat Latinas as a homogenous group that is experiencing similar stressors under the same circumstances [16, 17].

The current study aimed to evaluate the relationship between three dimensions of social support (e.g., informational, emotional, and instrumental) and perceived stress, postpartum distress, and depression in the 12 months after childbirth in Latinas. Last, we examine if these associations were moderated by nativity, years in the U.S., and language of preference.

## Methods

The Maternal and Developmental Risks from Environmental and Social Stressors (MADRES) study is an ongoing prospective cohort study of more than 1000 pregnant women focused on examining the psychosocial, behavioral, and environmental risk factors for maternal and infant health outcomes [18]. The MADRES cohort is composed of a primarily low-income Hispanic/Latina population of pregnant women in Los Angeles County. Participants were recruited from four prenatal care centers in Los Angeles beginning in 2015: one county hospital clinic, two non-profit community health clinics, and a private obstetrics and gynecology practice. To be eligible to participate in the larger study, participants had to be at least 18 years old, less than 30 weeks gestation at the time of recruitment, and a fluent Spanish or English speaker. Participants were not eligible if they were HIV positive, were incarcerated, had multiple gestations, and/or they reported any physical, mental, or cognitive disabilities that prevented participation/informed consent. The USC Institutional Review Board approved all study procedures. Data from a total of 137 mothers were drawn from the larger study. This subset of participants self-identified as Hispanic/Latina and completed a social support questionnaire at 1 month postpartum as well as a mental health outcome at 3, 6, or 12 months postpartum.

### Demographic Information

Demographic information was collected from a series of orally administered questionnaires during pregnancy which included data on maternal age, marital status (Married/living together, Never married, Divorced, Missing), childbearing history (Nulliparous, Primiparity/multiparity, Missing), household income (Less than $29,999, $30,000 to $100,000, Don't Know), and educational attainment (< 12th Grade, Completed 12th Grade, Some College or Technical School, Completed College, Some Graduate Training). Participants also self-reported their language preference (Spanish, English), country of birth (U.S., Mexico, Other Central America), and years living in the U.S. Language of preference was recorded at each time point that each study questionnaire was administered.

### Social Support Measures

Emotional, informational, and instrumental support were measured at one month postpartum using three 4-item PROMIS^®^ (Patient-Reported Outcomes Measurement Information System) scales for each individual domain [19]. Responses to each item were given on a 5-point Likert-type scale ranging from ‘never’ to ‘always’. Total raw scores ranged from 8 to 20. The raw scores were then converted to T scores (M = 50, SD = 10) in which higher scores indicate higher social support. Sample items included “I have someone who will listen to me when I need to talk (Emotional Support), “I have someone to give me good advice about a crisis if I need it” (Informational Support), and “Do you have someone to help you if you are confined to a bed?” (Instrumental Support).

### Postpartum Mental Health Outcomes

#### Perceived Stress

Perceived stress was measured using the 10-item Perceived Stress Scale [20]. Perceived stress was collected at 3, 6, and 12 months postpartum. The self-reported scale was designed to measure the degree to which participants’ lives are unpredictable, uncontrollable, and overloaded. Responses to each item were on a 0–4 Likert-type scale ranging from ‘never’ to ‘very often’. Sample item includes “In the last month, how often have you felt that you were unable to control the important things in your life?” Individual answers were totaled for an overall PSS score ranging from 0 to 40. Higher scores indicate higher levels of perceived stress experienced within the last month.

#### Postpartum Distress

We administered 9 items from the 10-item Postpartum Distress Measure (PDM) at 3, 6, and 12 months postpartum [21]. The PDM scale focuses on depressed mood as well as postpartum anxiety. Evidence indicates that the PDM is a helpful tool in identifying psychological distress as well as obsessive–compulsive symptoms experienced during postpartum [21]. Responses to each item were on a 0 to 3 Likert-type scale ranging from “No, this is not true” to “This is true most of the time”. Sample items include “I have recurring thoughts about my baby getting sick or having some kind of problem,” and “I check on my baby multiple times throughout the night.” Items were summed to create a total score ranging from 0 and 27. Higher scores indicate higher postpartum distress.

#### Probable Depression

The level of depressive symptoms at 12 months postpartum was obtained using the Center for Epidemiologic Studies Depression Scale (CES-D) [22]. The CES-D scale is a widely used, validated 20-item instrument that assesses depressive symptoms experienced within the past week. Example items include “I did not feel like eating; my appetite was poor” and “I felt that I could not shake off the blues even with help from my family or friends”. Responses to each item were on a 0 to 3 Likert type scale: ‘Rarely or None of the Time (Less than 1 Day)’ to ‘Most or all of the time (5–7 days)’. A total depressive symptomology score was calculated using the sum of the 20 items. Scores range from 0 to 60 with higher scores indicating higher depressive symptomology.

#### Statistical Analysis

Distributions of participant characteristics were summarized using means and standard deviations for continuous variables and frequencies and percentages for categorical variables. Linear regressions were used to assess the association between early postpartum social support and indicators of postpartum mental health across the first year after childbirth. To meet the assumptions of normality, variables with skewed distributions (postpartum distress at 3 months, postpartum distress at 6 months, and depressive symptoms at 12 months), were log-transformed. A constant of one was added to the data before applying the log transformation. Results were back-transformed for interpretation. We considered several sociodemographic variables as potential covariates based on directed acyclic graphs [23]. Final models were adjusted for covariates: recruitment site, age at recruitment, earliest ascertained total household income during pregnancy (Less than $29,999, $30,000 to $100,000, Don't Know), maternal marital status (Married/living together, Never married, Divorced, Missing), and parity (Nulliparous, Primiparity/multiparity, Missing).

To determine if acculturation factors modified these associations, we created a multiplicative interaction term between social support domains (continuous) and country of birth (categorical: U.S., Mexico, Other Central America), years in the U.S. (continuous; foreign-born Latinas only), and language of preference (binary) at the time of the outcome data collection. When examining the interactions between social support and years in the U.S. in foreign-born Latinas, U.S. born participants were excluded. For our continuous moderator, of years in the U.S, we chose to examine the levels at minimum, first quantile, median, 3rd quantiles, and maximum, (i.e., 0.16, 7.62, 15.08, 22.54, 30 years) for interpretability. If there was evidence of a significant interaction, we investigated simple slopes; we calculated the effect of the independent variable (social support domains) on stress and depression within the levels of the moderator (acculturation factor) [24]. Data management and analyses were conducted using SAS Version 9.4.

## Results

### Participant Characteristics

Participants were on average 28.4 (SD = 6.1) years old at the time of recruitment, foreign-born women had spent an average of 13.41 (SD = 7.73) years in the U.S, and 45% chose Spanish as the language of preference in over half of the data collection time points. Approximately 45% were born in the U.S. with approximately 31% being born in Mexico, and 22% were born in countries in Central America other than Mexico. Additional covariate information (e.g., income, marital status, parity distributions, and education status) is included in Table 1.

**Table 1:** Participant sociodemographic characteristics (n = 137)

Participant characteristic	n (%) or mean ± SD
Age at study entry	28.44 ± 6.11
Total household income	
Less than $29,999	54 (39.42)
$30,000–$100,000	23 (16.79)
Don’t know	60 (43.80)
Marital status	
Married/living together	100 (72.99)
Never married	24 (17.52)
Divorced	5 (3.65)
Missing	8 (5.84)
Parity	
Nulliparous	38 (27.74)
Primiparity/multiparity	97 (70.80)
Missing	2 (1.46)
Birthplace	
US-born Hispanic	62 (45.26)
Foreign-born Hispanic	73 (53.28)
Missing	2 (1.46)
Language preference	
English	75 (54.74)
Spanish	62 (45.26)
Education status	
< 12th grade	45 (32.85)
Completed 12th grade	43 (31.39)
Some college or technical school	35 (25.55)
Completed college	12 (8.76)
Some graduate training	2 (1.46)
Birth country	
U.S	62 (45.26)
Mexico	42 (30.66)
Other Central America	30 (21.90)
Missing	3 (2.18)
Years living in the U.S. (foreign-born Latinas only)	13.41 ± 7.73

### Associations Between Social Support Domains and Perceived Stress at 3, 6, and 12 Months

At three months postpartum, there were no significant associations between the social support domains and perceived stress (see Table 2). Results indicated significant associations between social support domains and perceived stress at 6 months postpartum. Specifically, higher reported emotional, informational, and instrumental support was associated with lower perceived stress. At 12 months postpartum, there was also a significant association between social support and perceived stress. Higher emotional support was associated with lower perceived stress. There were no significant associations between instrumental or informational support and perceived stress at 12 months postpartum.

**Table 2:** Associations between domains of social support and postpartum mental health outcomes

Postpartum outcome	Timepoint	Social support domain																	
		Instrumental						Emotional						Informational					
		Unadjusted B	SE	p	B^a^	SE	p	Unadjusted B	SE	p	B^a^	SE	p	Unadjusted B	SE	p	B^a^	SE	p
Perceived stress																			
	3 months	− 0.11	0.05	0.04	− 0.10	0.06	0.12	− 0.19	0.07	< 0.01	− 0.12	0.07	0.09	− 0.12	0.05	0.02	− 0.06	0.06	0.29
	6 months	− 0.20	0.06	< 0.001	− 0.21	0.06	< 0.001	− 0.23	0.07	< 0.01	− 0.21	0.07	0.01	− 0.15	0.06	0.01	− 0.13	0.06	0.03
	12 months	− 0.10	0.05	0.07	− 0.08	0.06	0.19	− 0.25	0.06	< 0.001	− 0.19	0.07	0.01	− 0.16	0.05	< 0.01	− 0.10	0.06	0.07
Postpartum distress																			
	3 months	− 0.02	0.01	0.21	− 0.01	0.01	0.15	− 0.02	0.01	0.02	− 0.01	0.01	0.11	− 0.01	0.01	0.09	− 0.01	0.01	0.23
	6 months	− 0.01	− 0.01	0.05	− 0.01	0.01	0.05	− 0.02	− 0.02	0.03	− 0.02	0.01	0.03	− 0.01	0.01	0.10	− 0.01	0.01	0.09
	12 months	0.00	0.04	0.9	− 0.03	0.04	0.50	− 0.06	0.04	0.18	− 0.07	0.05	0.16	0.00	0.04	0.96	− 0.01	0.04	0.71
Depression																			
	12 months	− 0.01	0.01	0.58	0.00	0.01	0.72	− 0.02	0.01	0.02	− 0.02	0.01	0.05	− 0.01	0.01	0.57	0.00	0.01	0.68

^a^Model adjusted for age, income, site, marital status, and parity

### Associations Between Social Support Domains Postpartum Distress at 3, 6, and 12 Months

Results indicated no significant relationships between the social support domains and postpartum distress at 3 months. At 6 months, there were also no significant associations between informational and instrumental support and postpartum distress. However, there was a significant association between emotional support and postpartum distress at 6 months. Higher emotional support was associated with lower postpartum distress. Last, there was no significant association between any of the social support domains and postpartum distress at 12 months postpartum.

### Associations Between Social Support Domains and Depression at 12 Months

Results indicated no significant associations between any of the social support domains and depression at 12 months postpartum.

### Effect Moderation of Associations by Preferred Language

We evaluated whether the relationships between social support domains and measures of mental health were moderated by mothers’ preferred language at the time of data collection (e.g., 3, 6, and 12 months postpartum) (see Supplemental Materials: Table 1). There was no indication that the association between informational, emotional, or instrumental support and perceived stress postpartum was moderated by participants’ preferred language.

**Fig. 1:**
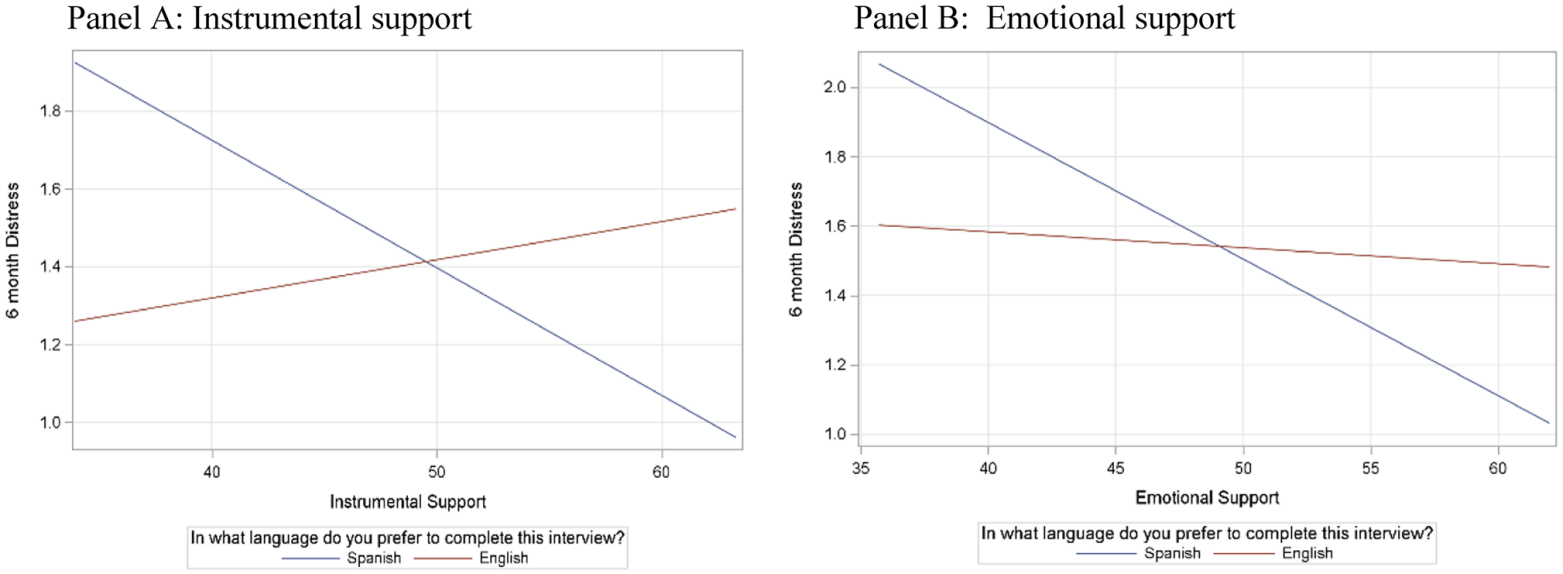
Moderation of associations between social support domains and postpartum distress at 6 months by language of preference

We also evaluated whether the associations between social support domains and postpartum distress were moderated by preferred language at 3, 6, and 12 months postpartum. We found that language preference moderated the association between instrumental support and postpartum distress at 6 months (*p interaction* < 0.01) (see Fig. 1: panel A) such that for mothers who preferred to speak Spanish, higher instrumental support was associated with lower postpartum distress. We conducted simple slope tests to examine the association between instrumental support and postpartum distress for each language of preference. There was a significant negative association between instrumental support and postpartum distress for mothers whose language of preference was Spanish, B = − 0.03, p < 0.001, CI [− 0.05, − 0.01]. There was no significant association between instrumental support and postpartum distress for mothers whose language of preference was English, B = 0.13, p = 0.37, CI [− 0.01, 0.03].

We also found a significant interaction between emotional support and language associated with postpartum distress at 6 months (*p interaction* = 0.04) (see Fig. 1: panel B) such that for mothers who preferred to speak Spanish, higher emotional support was associated with lower postpartum distress. Simple slope tests showed that the association was significant for those that preferred Spanish, B = − 0.04, p = 0.002, CI [− 0.06, − 0.01]. The association was not significant for those that preferred English, B = − 0.004, p = 0.69, CI [− 0.03, 0.02]. At 12 months postpartum, there were no significant interactions between any of the social support domains and preferred language predicting postpartum distress. Lastly, the relationships between social support domains and depression at 12 months were not significantly moderated by preferred language.

### Moderation of Associations by Years in the U.S.

We explored whether the number of years foreign-born Latina mothers had lived in the U.S. modified the associations between social support and measures of mental health. There was no evidence that years spent in the U.S. moderated the associations between social support domains and perceived stress at 3, 6, or 12 months postpartum. At 3 months postpartum, we observed that the relationship between emotional support and postpartum distress was significantly moderated by years spent in the U.S. (*p interaction* = 0.04) (see Supplemental Material: Fig. 1). Simple slopes tests showed that the association was significant for those that had spent less than 0.16 years in the U.S., B = − 0.04, p = 0.02, CI [− 0.08, − 0.01], such that for mothers with less than one year living in the U.S., higher emotional support was associated with lower postpartum distress at 3 months. The association was not significant for those that had spent 7.62 years, B = − 0.02, p = 0.11, CI [− 0.04, 0.00], 15.08 years, B = 0.00, p = 0.77, CI [− 0.02, 0.03], 22.54 years, B = 0.03, p = 0.22, CI [− 0.02, 0.07], and 30 years in the U.S., B = 0.05, p = 0.12, CI [− 0.01, 0.11].

**Fig. 2:**
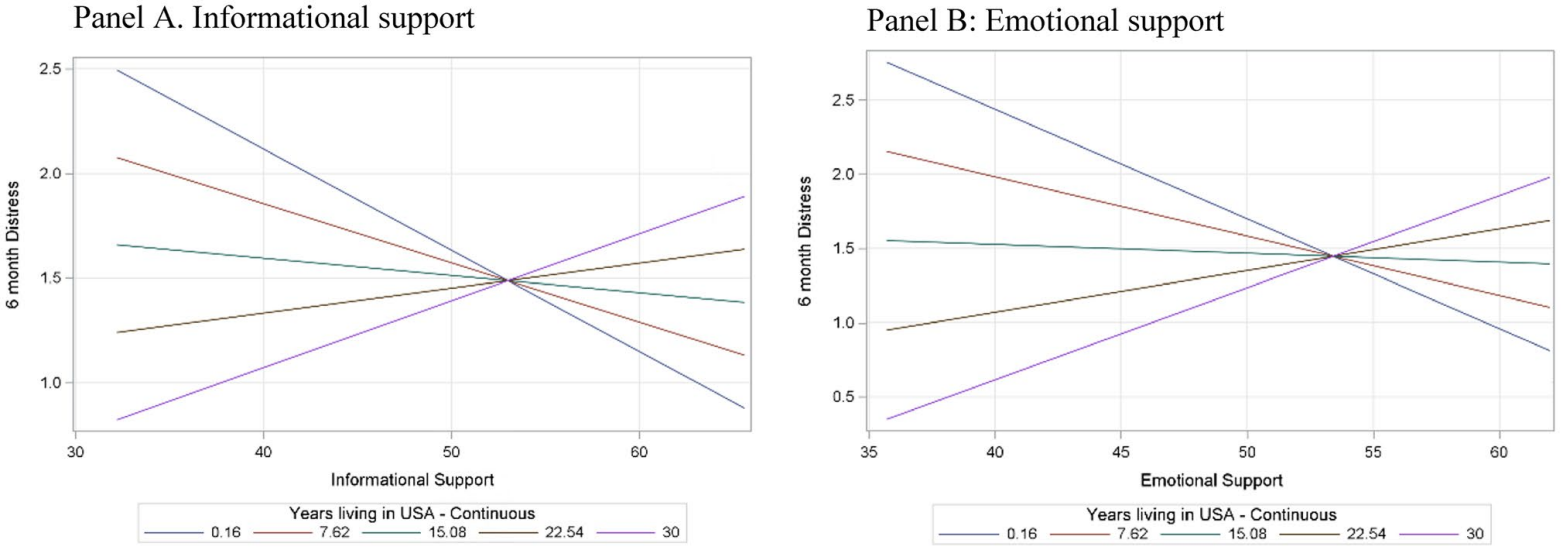
Moderation of associations between social support domains and postpartum distress at 6 months by years living in the US

At 6 months postpartum, we also observed that the relationship between informational support and postpartum distress at 6 months was moderated by years spent in the U.S. (*p interaction* = 0.03) (see Fig. 2: panel A) such that for mothers who had spent less than eight years in the U.S., higher informational support was associated with lower postpartum distress at 6 months. Simple slope tests showed that the association was significant for those that had spent 0.16 years, B = − 0.05, p < 0.01, CI [− 0.08, − 0.02], and 7.62 years in the U.S., B = − 0.03, p = 0.001, CI [− 0.05, − 0.01].

The association was not significant for those that had spent 15.08 years, B = − 0.01, p = 0.45, CI [− 0.03, 0.01], 22.54 years, B = 0.01, p = 0.47, CI [− 0.02, 0.01], and 30 years in the U.S., B = 0.03, p = 0.20, CI [− 0.02, 0.08].

Similarly, the association between emotional support and postpartum distress was moderated by years in the U.S. at 6 months (*p interaction* < 0.01) (see Fig. 2: panel B). Simple slope tests showed that the association was significant for those that had spent 0.16 years, B = − 0.07, p < 0.001, CI [− 0.11, − 0.04], and 7.62 years in the U.S., B = − 0.04, p < 0.01, CI [− 0.06, − 0.02].

The association was not significant for those that had spent 15.08 years, B = − 0.01, p = 0.65, CI [− 0.03, 0.02], 22.54 years, B = 0.03, p = 0.20, CI [− 0.01, 0.07], and 30 years in the U.S., B = 0.06, p = 0.05, CI [0.00, 0.13].

At 12 months postpartum, we found a significant interaction between instrumental support and years in the U.S. predicting probable depression at 12 months postpartum (*p interaction* = 0.03) (see Supplemental Material: Fig. 2) such that for mothers who had spent one year in the U.S., higher instrumental support was associated with lower postpartum depressive symptoms at 12 months. Simple slope tests showed that the association was significant for those that had spent 0.16 years, B = − 0.05, p = 0.04, CI [− 0.09, 0.00]. The association was not significant for those that had spent 7.62 years in the U.S., B = − 0.02, p = 0.21, CI [− 0.05, 0.01], 15.08 years, B = 0.01, p = 0.55, CI [− 0.02, 0.04], 22.54 years, B = 0.04, p = 0.14, CI [− 0.01, 0.09], and 30 years in the U.S., B = 0.06, p = 0.08, CI [− 0.01, 0.14].

### Effect Moderation of Associations by Country of Birth

We explored whether mothers’ country of birth modified the associations between social support and measures of mental health. We found no indication that country of birth moderated any of the associations between social support scales and perceived stress at 3, 6, or 12 months postpartum. We also found that country of birth did not significantly modify the associations between social support domains and postpartum distress at 6 months.

**Fig. 3:**
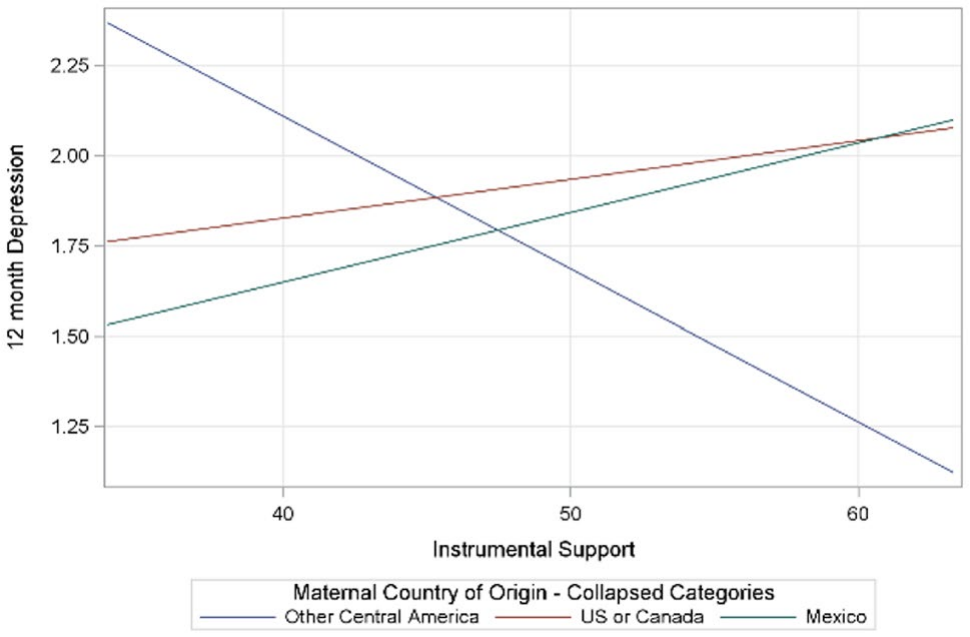
Moderation of associations between instrumental support and depression at 12 months by country of birth

Country of birth did significantly moderate the association between instrumental support and depression at 12 months postpartum (*p interaction* = 0.01) (see Fig. 3) such that for mothers born in Central American countries, higher instrumental support was associated with lower depressive symptoms at 12 months postpartum. Simple slope tests showed that the association was significant for those from Central American countries, B = − 0.04, p = 0.01, CI [− 0.08, − 0.01] but not US, B = 0.01, p = 0.52, CI [− 0.02, 0.04], or Mexico, B = 0.02, p = 0.30, CI [− 0.02, 0.06].

## Discussion

We examined the association between perceived social support and postpartum mental health (i.e., perceived stress, postpartum distress, depression) as well as moderations by acculturation factors in a sample of primarily low-income U.S. and foreign-born Latina women in Los Angeles. Results indicated that greater perceived social support, measured at 1 month postpartum, was associated with lower perceived stress at 6 and 12 months postpartum. Specifically, perceived informational, emotional, and instrumental support were each inversely related to perceived stress at 6 months postpartum. Higher emotional support was associated with lower levels of perceived stress at 12 months postpartum as well as postpartum distress at 6 months postpartum.

Few studies have examined the relationships between social support domains and maternal mental health in specifically low-income Latina samples. Nevertheless, our results are consistent with extant work showing an inverse relationship between social support and perceived stress in postpartum Latinas. According to Mann et al. [11], social support was shown to be a protective factor for perceived stress in both English and Spanish reading postpartum Latina women. Our results are also consistent with Pao et al. [12] which indicates that the presence of social support may buffer against postpartum depression among racial and ethnic minority women.

Our results are also consistent with previous findings based on non-Latino samples. According to Reid and Taylor [25] analysis of the Fragile Families and Child Well-being Study, higher levels of perceived social support decreased the risk for depressive symptoms among postpartum mothers. While our study did not investigate whether social support served as a moderator between perceived stress and depression, Reid and Taylor [25] found no evidence that social support buffered against stress during the postnatal period. Our results are also in line with Leahy-Warren et al.’s [8] findings; they found distinctive associations of emotional and functional support with postnatal depression at 6 and 12 months postpartum in a sample of mothers recruited in the Republic of Ireland. Specifically, only lower functional support versus emotional support was associated with higher depressive symptoms in the postnatal period.

We also examined the interactions between perceived social support and preferred language, country of birth, and years in the U.S. predicting maternal mental health outcomes. There was evidence that acculturation factors moderated the relationship between social support domains and mental health. Specifically, foreign-born Latina mothers that had spent fewer years in the United States had a stronger inverse relationship between social support and mental health outcomes. These findings are consistent with studies showing that more time spent in the U.S. is linked with worse health outcomes among foreign-born Latinas [26]. Mothers who were born in Central American countries had a significant association between higher social support and better mental health outcomes, and mothers whose language of preference was Spanish compared to English also had a stronger inverse relationship between social support and mental health outcomes. This finding is in contrast to a study that showed that the association between social support and perceived stress did not differ between mothers that speak English versus Spanish [11], suggesting that women who are more acculturated may benefit less from social support.

A possible reason that we did not find moderation effects across all acculturation factors was that we did not distinguish between the available sources (i.e., different types of people/relationships) of social support. The PROMIS social support scale measures the perception of the availability or adequacy of resources provided by others in the social network. Previous studies indicate that postpartum women are more likely to identify immediate family members versus friends as principal sources of instrumental support [27]. According to Reid and Taylor [25], support from an intimate partner compared to support from friends and family members was more beneficial for married women.

Family social support sources are particularly important to study in U.S. Latinas because they place a strong value on familial relationships [28]. Higher levels of familism, a core cultural value characteristic of Latino individuals that emphasizes close, warm, and supportive, relationships, have been associated with less anxiety, depression, and perceived stress [10, 28]. Future studies should examine whether social support perceived from familial sources or stronger familism values reduce the risk of postpartum mental health distress. It is possible that women who are more acculturated may have weaker family ties, which may result in less beneficial social support.

A strength of our study is the inclusion of an often medically underserved and under-researched population. Foreign and U.S.-born Latinas experience stressors related to immigration and socioeconomic inequities that may strongly affect their postpartum psychological health compared to other U.S. subpopulations. On the other hand, while our sample size was small, findings from the MADRES pregnancy cohort will be generalizable to vulnerable foreign, and U.S.-born Latina populations. The most important strength of our study was that we distinguish the subtypes of perceived social support measured. This paper signifies the importance of considering the contributions of each social support dimension and their prediction of maternal health and well-being.

A limitation of our study is that because this was a secondary research question, we were limited by the extant data available. Given the small sample size, we explored acculturation factors independent of each other increasing the risk of type 1 error. We did not have the power to explore the intersectionality of the Latina postpartum mother experience; it would be beneficial to explore how the interplay of social support and acculturation factors influence psychological distress. For instance, would Latina mothers who were born in Mexico and have spent fewer years in the U.S. have the same experience as those born in Guatemala and have been in the U.S. for decades? In addition, the measures used in this study (i.e., the language of preference, years in the U.S., and country of birth) were proxies for acculturation. It is important to clearly define and measure acculturation to understand how culture, values, and belief systems influence health and healthcare interventions [29].

Given our Latina-specific sample, there is a need to examine whether the relationships between social support and maternal mental health we found are exacerbated by cultural stressors especially in those who are less acculturated. Latina mothers are highly vulnerable to psychosocial stressors such as acculturative stress, discrimination, and economic hardships that are predictive of psychological distress [29]. It is possible that emotional, instrumental, or informational support may have ceiling effects and not serve as a robust protective factor in the face of additional psychosocial barriers.

In conclusion, there is evidence that social support serves as a protective factor against adverse mental health in the first postpartum year among a sample of low-income Latinas who are less acculturated. These findings demonstrate the importance of examining culturally relevant, psychosocial factors such as social support that influence Latina maternal health. Furthermore, this is one of the few studies that distinguish the influence of three dimensions (emotional, instrumental, and informational) of social support. Our findings shed light on the need to continue to study various parameters of mental health status (i.e., perceived stress, postpartum distress, depression) at different time points during the postpartum period. This work may ultimately inform interventions aimed at increasing social support and mental health for a heterogenous group of Latinas living in the U.S.

### Supplementary Information

Below is the link to the electronic supplementary material.

Supplementary file1 (DOCX 433 kb)
