# Supplementary material for: The Role of Social Support and Acculturation Factors on Postpartum Mental Health Among Latinas in the MADRES Pregnancy Cohort
Source: J Immigr Minor Health. 2023 Oct 28;26(1):72–80. doi: 10.1007/s10903-023-01542-w (PMC10771371; doi:10.1007/s10903-023-01542-w)

Supplemental Material


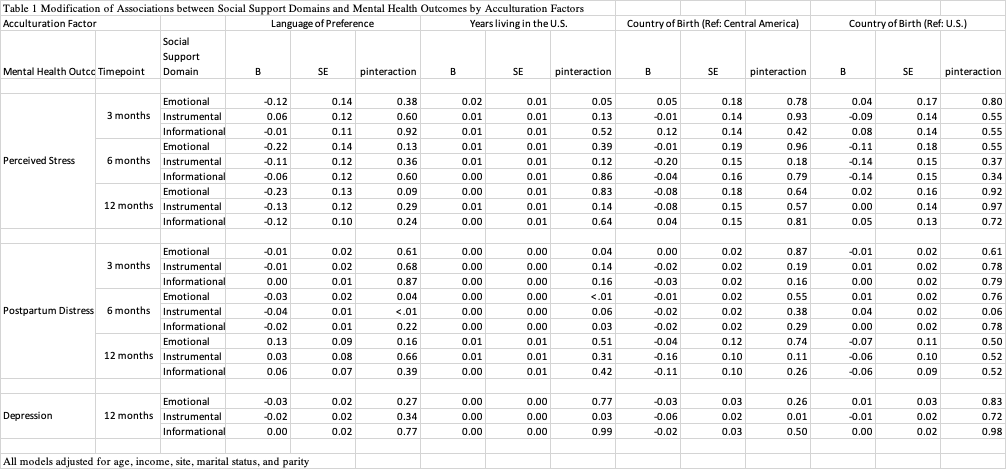


Figure 1. Emotional support and years in the U.S. association with distress at 3 months postpartum

**
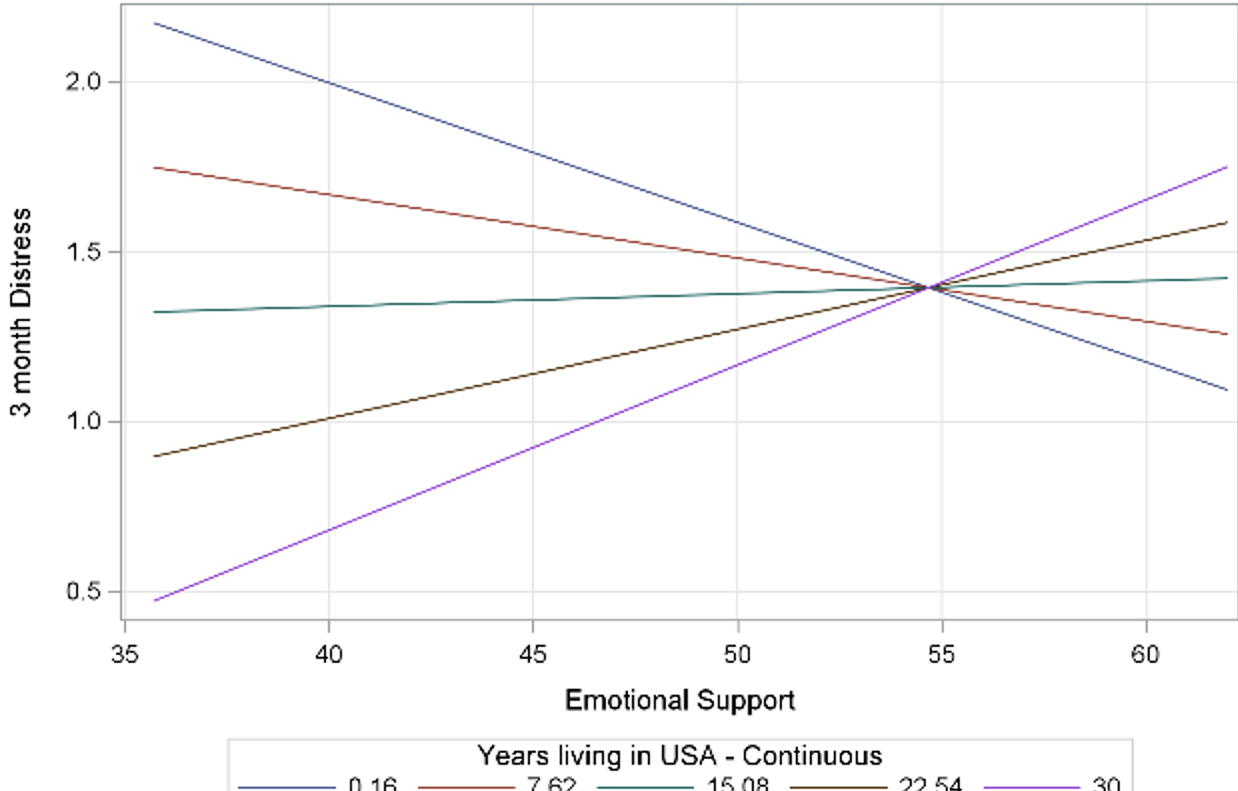
**

Figure 2. Instrumental support and years in the U.S. association with depression at 12 months postpartum


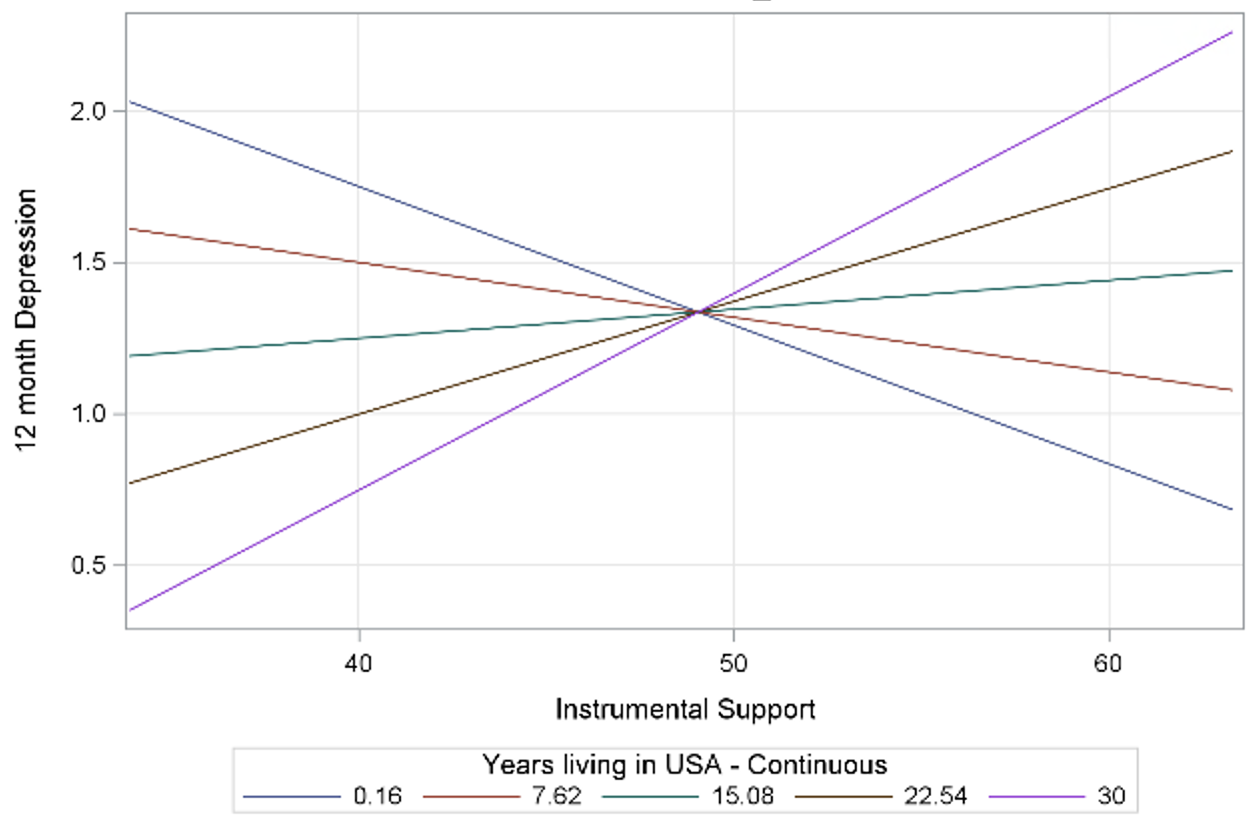

Supplement: Supplementary file 1 — Supplementary file1 (DOCX 433 kb) [file 10903_2023_1542_MOESM1_ESM.docx]
